# *In vitro* maturation medium supplementation: utilization of repaglinide, L-carnitine, and mesenchymal stem cell-conditioned medium to improve developmental competence of oocytes derived from endometriosis mouse models

**DOI:** 10.1590/1414-431X2022e11948

**Published:** 2022-04-27

**Authors:** E. Kalehoei, M. Moradi, M. Azadbakht, H. Zhaleh, M. Parvini, S. Cheraghbaeigi, S. Saghari

**Affiliations:** 1 Department of Biology, Faculty of Basic Sciences, Razi University, Kermanshah, Iran; 2 Department of Clinical Sciences, Faculty of Veterinary Medicine, Razi University, Kermanshah, Iran; 3 Fertility and Infertility Research Center, Health Technology Institute, Kermanshah University of Medical Sciences, Kermanshah, Iran; 4 Substance Abuse Prevention Research Center, Health Institute, Kermanshah University of Medical Sciences, Kermanshah, Iran

**Keywords:** Endometriosis, Oocyte quality, Maturation culture medium, Bone marrow mesenchymal stem cell, Oxidative stress, Female infertility

## Abstract

Endometriosis (EMS) is one of the most prevalent causes for female infertility. Herein, we investigated the effect of the repaglinide (RG), L-carnitine (LC), and bone marrow mesenchymal stem cell-conditioned medium (BMSC-CM) supplementation during *in vitro* maturation (IVM) on the quality, maturation, and fertilization rates, as well as embryonic quality and development of oocytes derived from normal and EMS mouse model. Immature oocytes were collected from two groups of normal and EMS-induced female NMRI mice at 6-8 weeks of age. Oocytes were cultured in IVM medium unsupplemented (control group), or supplemented with 1 M RG, 0.3 and 0.6 mg/mL LC, and 25 and 50% BMSC-CM. After 24 h of oocyte incubation, IVM rate and antioxidant status were assessed. Subsequently, the rates of fertilization, cleavage, blastulation, and embryonic development were assessed. Our results demonstrated that supplementation of IVM medium with LC and BMSC-CM, especially 50% BMSC-CM, significantly enhanced IVM and fertilization rates, and markedly improved blastocyst development and total blastocyst cell numbers in EMS-induced mice compared to the control group (53.28±0.24 *vs* 18.09±0.10%). Additionally, LC and BMSC-CM were able to significantly modulate EMS-induced nitro-oxidative stress by boosting total antioxidant capacity (TAC) and mitigating nitric oxide (NO) levels. Collectively, LC and BMSC-CM supplementation improved oocyte quality and IVM rates, pre-implantation developmental competence of oocytes after *in vitro* fertilization, and enhanced total blastocyst cell numbers probably by attenuating nitro-oxidative stress and accelerating nuclear maturation of oocytes. These outcomes may provide novel approaches to refining the IVM conditions that can advance the efficiency of assisted reproductive technologies in infertile couples.

## Introduction

Endometriosis (EMS) is a detrimental condition of the female reproductive system in which the endometrium (uterine lining) grows outside the uterus, most commonly on the ovary and peritoneum. The main symptoms of the disease are pelvic pain, dysmenorrhea, and dyspareunia (1). In addition to the fact that EMS impacts up to 15% of women of reproductive age, 25-40% of women with infertility have been estimated to suffer from EMS (2). However, the exact pathophysiology of EMS related to infertility is still unknown. It can be detrimental to fertility directly by distorting tubo-ovarian anatomy or indirectly by invoking inflammatory and oxidative damage to the oocytes resulting in poorer quality oocytes (3,4). In addition, EMS is currently believed to be detrimental to the ovaries based on molecular, histological, and morphological evidence (5).

Over the past three decades, assisted reproductive technology (ARTs) has encompassed the management of almost all types of infertility, including EMS. In this context, recent studies have shown that oocytes retrieved from EMS-affected ovaries are more likely to fail *in vitro* maturation (IVM) and to show altered morphology and lower cytoplasmic mitochondrial content (3). Moreover, oocyte quality is reflected in the ability of the cell to complete maturation and undergo successful fertilization and plays a vital role in embryonic development during fertilization (3). The available evidence suggests that a reduction in the quality of oocytes retrieved is consistently associated with EMS, differently than other causes of infertility (5). In essence, EMS has a negative impact not only on the receptivity of the endometrium but also on the development of oocytes and embryogenesis (3). However, human oocytes are relatively rare for research, and their use in invasive investigations is typically unviable because it prevents their use in ARTs. On this premise, animal models may be beneficial in elucidating the pathophysiology of EMS-associated infertility.

Nitro-oxidative stress is a condition that reflects an imbalance between the systemic manifestation of reactive oxygen species (ROS) and reactive nitrogen species (NOS) and the ability of a biological system to readily detoxify the reactive intermediates or to repair the resulting damage (6). Oxidative stress may have detrimental effects on oocytes, the fertilization process, and subsequent embryo development (3,4). Recent studies have demonstrated that free radicals play a critical role in the pathophysiology of EMS (7). Moreover, the follicular fluid of patients with EMS shows increased levels of reactive species and a reduction in total antioxidant capacity (4,7).

Repaglinide (RG) is an oral anti-hyperglycemic medication used to treat non-insulin-dependent diabetes mellitus. It belongs to the meglitinide class of short-acting insulin secretagogues, which induce insulin secretion by attaching to the β cells of the pancreas (8). RG achieves this by inhibition of the K-ATP-sensitive channels in the membrane of the β cells (9). This depolarizes the β cells, allowing voltage-gated calcium channels to open, and the subsequent calcium influx stimulates insulin release (10). It has been reported that RG could up-regulate glutathione reductase and glutathione levels, thereby enhancing the anti-oxidative defenses (11). While the potential of RG in treating diabetes has been investigated well, there is little information to support its effect on oocyte maturation and subsequent developmental process.

L-carnitine (LC; β-hydroxy-c-trimethylammonium-butyric acid) is a vital cofactor that may be generated endogenously or received through dietary sources and plays an important role in cell metabolism (12). LC is crucial for fatty acid metabolism because it facilitates the transport of long-chain free fatty acids into the mitochondrial matrix, where they may be used for beta-oxidation (12,13). Furthermore, LC transports acetyl groups from the inside to the outside of the mitochondrial membrane, regulating glucose metabolism and, as a result, affecting cell ATP levels (13). LC also possesses direct antioxidant properties, preserves mitochondrial metabolism, and suppresses ROS-producing enzyme activities (14). Beneficial effects of LC on embryonic development in culture have been observed in many mammalian species (15). In mice, supplementation of the IVM medium with LC promotes spindle microtubule assembly and chromosome alignment in MII oocytes and improves subsequent embryonic development by preventing apoptosis (15). Oocyte metabolism is linked to oocyte quality, and it was recently discovered that beta-oxidation of lipids is required for oocyte developmental competence (16).

Mesenchymal stem cells (MSCs) are adult and multipotent stem cells with self-renewal capacity that can develop into cells of numerous unique mesodermal lineages, including bone, cartilage, and adipose tissues (17). According to various studies, MSCs secrete various types of cytokines, growth factors, bioactive factors, and tissue regenerative components into mesenchymal stem cell-conditioned medium (MSC-CM) (17,18). Moreover, MSCs release anti-apoptotic molecules, including Bcl-xL and Bcl-2, as well as antioxidant proteins like peroxiredoxin-5 (PRDX5) (17). Since cytokines and growth factors are known to enhance meiotic progression and the processes involved with IVM (17,18), we examined whether IVM, *in vitro* fertilization (IVF), and subsequent embryonic processes with oocytes derived from EMS-induced mice could be improved by MSC-CM.

Hence, the present study was aimed to investigate the comparative effect of RP, LC, and bone marrow MSC-CM (BMSC-CM) supplementation during IVM on the developmental competence of oocytes derived from normal and EMS-induced mice in terms of IVM, IVF, and subsequent developmental rate, as well as on the TAC and NO levels in the IVM medium.

## Material and Methods

### Material

All chemicals were purchased from Sigma Chemical Corporation (USA) and Gibco (USA), except repaglinide that was purchased from Farabi Corporation (Iran).

### Animals and experimental design

Adult female NMRI mice (6-8 weeks old) were purchased from Pasteur Institute (Iran). The animals were first habituated for one week and then divided into control and experimental groups. The animals were held under standard conditions (12-h light-dark cycles, 23±1°C, and 50-60% humidity) and had *ad libitum* access to water and food (standard diet) throughout the study. All experimental procedures pursued international guidelines for the care and use of laboratory animals and were approved by the Animal Welfare and Ethics Committee of Basic Sciences, Razi University, Kermanshah, Iran. The studied groups included two normal mice and mice under EMS induction. Oocytes obtained from normal mice were cultured in the IVM medium supplemented with RG, LC, and BMSC-CM. Likewise, oocytes derived from EMS-induced mice were cultured in the IVM medium containing RG, LC, and BMSC-CM.

### Endometriosis induction

Two groups of mice (6-8 weeks old) were used to induce EMS. To establish the EMS model, the mice in the donor group were intraperitoneally injected with estradiol-17β depot diluted in sesame oil (100 μg/kg) for one week. Then, they were sacrificed on day 14, and their uterine horns were removed. In the next step, tissue fragments from both uterine horns were harvested in a petri dish containing warm sterile saline. The provided suspension was injected intraperitoneally to the mice of the recipient group (approximately 40-50 fragments per mouse) according to Somigliana et al. (19) method with some modification.

### Culture of mesenchymal stem cells and collection of conditioned medium

Bone marrow mesenchymal stem cells (BMSCs) were isolated from 6-8-week old NMRI mice. Briefly, bone marrow was harvested by flushing femurs and tibias that were cultured in Dulbecco's Modified Eagles Medium (DMEM; Gibco, USA) consisting of 10% fetal bovine serum (FBS), L-glutamine 2 mM, 1% non-essential amino acids, and 1% penicillin/streptomycin (incubation at 37°C and 5% CO_2_). After 3 days of culture, non-adherent cells were removed by washing twice with PBS, and culture of adherent cells continued for 5-7 days until 80% confluence; the medium was changed every 2-3 days. At the third passage, the cells were trypsinized and seeded at a density of 1×10^4^ cells/cm^2^ in a culture flask. After reaching 80% confluence to prepare a conditioned medium of BMSCs, the cells were washed three times with PBS and incubated for 48 h at 37°C and 5% CO_2_ in a serum‐free DMEM culture medium. After 48 h of incubation, the supernatant (conditioned medium) was collected and filtered through a 0.2-μm filter for immediate use. The BMSCs cells were derived from female and male NMRI mice. For detection, we used flow cytometry for CD14, CD45, CD34, CD73, CD90, CD105, and CD 29 to detect the phenotype of the 5th passage cells. The results showed that about 98% of BMSCs were CD90-positive and lacked expression of CD14, CD45, and CD34. These results showed that mouse bone marrow cells had the characteristics of mesenchymal stem cells (Supplementary Figure S1). The results have already been published in our previous article (20).

### Histological examination of ovaries

**Figure 1:**
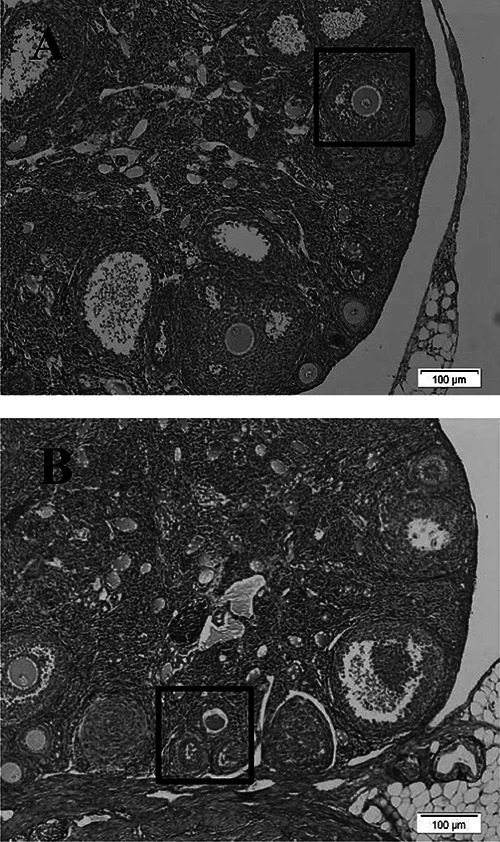
Histological comparison of normal (**A**) and endometriosis-induced (**B**) ovaries of mice (scale bar: 100 μm). **A**, boxed area shows healthy growing follicle. **B**, boxed area shows atretic follicles.

In order to ensure the induction of EMS and its impacts on the ovaries, some mice in both normal and EMS groups were randomly selected, and after sacrifice, their ovaries were fixed in Bouin's solution, embedded in paraffin wax, and serially sectioned at 5 μm. Then, the serial sections of ovaries were stained with hematoxylin and eosin (HE). Afterward, the diagnosis of EMS was determined under a light microscope according to the morphological criteria such as different stages of follicular growth (folliculogenesis) and follicular quality, dead or atretic follicles, changes of oocyte quality, presence of residual cyst, and bleeding in the ovarian tissue (Figure 1).

### Collection of oocytes and IVM

**Figure 2:**
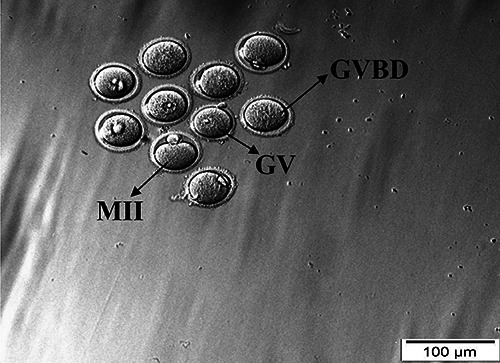
Different stages of *in vitro* maturation of mice oocytes (scale bar: 100 μm). GV: germinal vesicle; GVBD: germinal vesicle breakdown; MII: metaphase II.

Female NMRI mice from normal and EMS groups were sacrificed by an inhaled overdose of carbon dioxide (CO_2_, 10-30%), followed by cervical dislocation (all efforts were made to minimize suffering). Then, their ovaries were removed and immediately transferred to the dissection medium of Alpha Minimal Essential Medium (α-MEM) containing 5% FBS and 1% penicillin/streptomycin. The immature oocytes (GV stage) were mechanically isolated from ovaries under a stereomicroscope (Motic: SMZ-143, China at 10× magnification) in 50-μL micro drops of dissection medium by using a 27-gauge needle. After washing three times with droplets of dissection medium by mouth pipette, GV oocytes were transferred into 30-μL drops of IVM medium consisting of α-MEM, supplemented with 4 mg/mL bovine serum albumin (BSA), 10 ng/mL recombinant epidermal growth factor (rEGF), 7.5 IU/mL human chorionic gonadotropin (HCG), and 100 IU/mL penicillin and 100 μg/mL streptomycin (in mineral oil at 37°C and 5% CO_2_). In both normal and EMS groups, experimental groups included control (IVM medium alone) and treatments (IVM medium supplemented by 1 µM RG, 0.3 and 0.6 mg/ml LC, and 25, 50% BMSC-CM). After 24-h incubation, IVM rate was assessed under an inverted microscope (Olympus, Japan) according to the observation of different stages of maturation such as germinal vesicle (GV), germinal vesicle breakdown (GVBD), metaphase II (MII), and degenerated (Deg) oocytes (Figure 2).

### Assessment of total antioxidant capacity (TAC) levels and NO levels

Twenty-four hours after incubation of oocytes, the IVM condition media from all experimental groups were collected and antioxidant capacity (TAC, NO levels) was assessed (21).

Spectrophotometer analysis with a colorimetric assay kit (Naxifer™, Navand Salamat Co., Iran) was used to estimate the concentrations of testicular levels of ferric reducing antioxidant power (FRAP). This procedure is based on the ability of testis lysis to reduce iron III (Fe^3+^) to iron II (Fe^2+^) in the presence of 2,4,6-tripyridyl-S-triazine (TPTZ). A complex with blue color and maximum absorbance appeared in 593 nm with a reaction of Fe^2+^ and TPTZ. Finally, the values are shown as nanomoles of Fe^2+^ equivalents per wet tissue weight (nmol/mg protein) (22).

The total NO content of the homogenized testis was measured according to the Griess reaction using the Natrix™ assay kit (Navand Salamat Co.). In the Griess reaction, NO rapidly converts into nitrite, which is an acidic environment, and then converts into HNO_2_. After adding sulfanilamide, HNO_2_ forms a diazonium salt that reacts with N-(1-Naphthyl) ethylenediamine dihydrochloride to form an azo dye, which can be measured at 570 nm. The NO content of the examined organs was reported in nmol/mg protein in samples (22).

### *In vitro* fertilization and embryo formation

**Figure 3:**
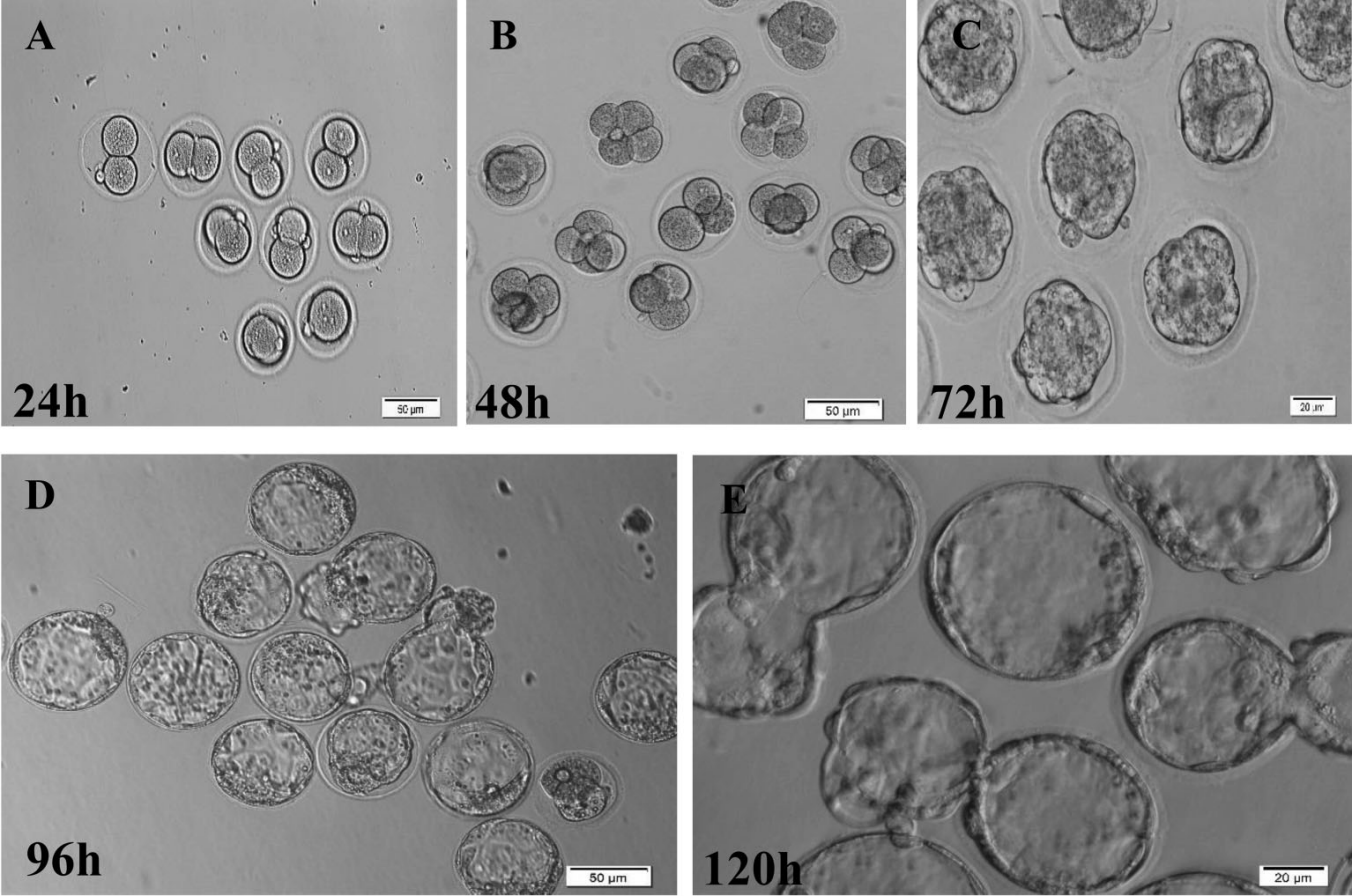
Different stages of mouse embryo cleavage at different times of *in vitro* culture [scale bar: 50 μm (A, B, D), 20 μm (C, E)]. **A**, 2 cells; **B**, 4 cells; **C**, Morula; **D**, expanded blastocysts; **E**, hatching blastocysts.

*In vitro* matured oocytes (MII) were transferred to the 50-μL drops of global IVF medium supplemented with 16 mg/mL of BSA. The cauda epididymis was isolated from 8-12-week-old male NMRI mice, and motile sperm fraction was obtained by the swim-up technique after a 45-min incubation at 37°C and 5% CO_2_ in the Ham's F10 medium containing 16 mg/mL of BSA, 10 μL of motile sperm (final concentration of 1×10^6^) added to each drop of IVF medium. After 4-6 h of sperm-oocyte incubation, the resulting zygotes were removed and washed three times in 50-μL drops of global medium with 4 mg/mL of BSA and subsequently transferred to the 30-μL drops of culture medium consisting of global media with 4 mg/mL of BSA in groups of 10 zygotes/drops that were covered with mineral oil (incubation at 37°C and 5% CO_2_). The embryos cleavage rates were assessed under an inverted microscope (Olympus, IX71) for 1 to 5 days, post-IVF (method described by Giritharan et al. (23) with some modification) (Figure 3).

### Blastocyst quality evaluation

**Figure 4:**
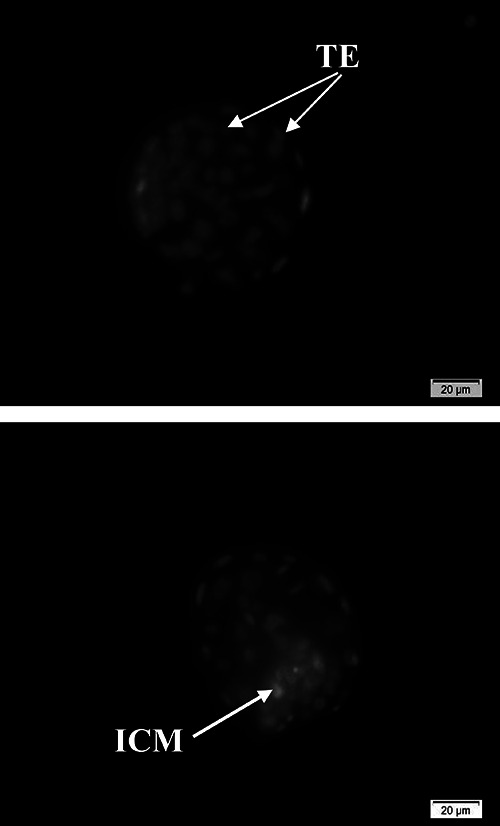
Differential staining of mice blastocyst after 5 days of *in vitro* culture (scale bar: 20 μm). ICM: inner cell mass, stained with propidium iodide would appear red. TE: trophectoderm, nuclei labeled with Hoechst 33258 would appear blue. Red and blue do not appear because these images are in black and white.

Differential staining was used to count the number of blastocysts and trophectoderms (TE) and assess the inner cell mass (ICM) of cells. At 96 h of embryo culture, blastocysts were washed several times in phosphate buffered saline (PBS, pH 7.2) and incubated at 37°C and 5% CO_2_ for 30 s in 500 µL of 100 µg/mL of propidium iodide (PI, Sigma) and 1% Triton X-100, then washed with PBS and transferred in 500 µL of absolute ethanol containing 25 µg/mL bisbenzamide (Hoechst 33258; Sigma) and incubated for 30 min at 37°C. Fixed and stained blastocysts were mounted in glycerol and observed under an inverted fluorescence microscope (Olympus IX71), and observed using UV light. The nuclei of TE cells labeled by Hoechst 33258 have a blue color and the nuclei of ICM cells labeled by propidium iodide have a red color. Finally, the quality of blastocysts was evaluated based on the ICM and TE cells (24) (Figure 4).

### Statistical analysis

Data analysis was done using the SPSS statistical software (version 19: SPSS Inc., USA). Normality and homogeneity of data were determined by Kolmogorov-Smirnov test. IVM, IVF, and embryo cleavage rates were analyzed by the chi-squared test. The TAC and NO levels and blastocyst cell numbers were evaluated by one-way ANOVA and Tukey's *post hoc* test. Quantitative data are reported as means±SE and 95% confidence interval (CI). Differences were considered statistically significant when the P-value was ≤0.05.

## Results

### Qualitative histological examination of normal and endometriosis ovaries

Microscopic studies of random ovarian tissue samples showed that folliculogenesis, quality of follicles, and number and quality of intact oocytes (atretic follicles or oocytes) were reduced in the EMS-induced group compared with the normal group. Also, the number of ovarian cysts, bleeding in the ovary, and adhesion of the ovary to the surrounding tissues and pelvic organs were increased. Consequently, all these factors led to a noticeable reduction in the ovulation rate.

### Evaluation of *in vitro* maturation of oocytes

Table 1 shows the characteristics of the different stages of oocyte maturation in the normal group. The percentages indicate that there was a significant difference between control and all treatment groups. Indeed, the highest IVM rate in both the normal and EMS mice was observed in the 50% BMSC-CM group. In the normal group, only 0.6 mg/mL LC and 25 and 50% BMSC-CM were able to considerably reduce the GV rate compared to the control group.

**Table 1:** *In vitro* maturation stages of immature oocytes derived from normal mice ovaries and from mice with endometriosis ovaries.

Groups	N	GV (%)	GVBD (%)	MII (%)	DEG (%)
Normal					
Co	112	16.48±1.07^b,c,d,e,f^	21.03±0.85^c,d,e,f^	55.16±1.03^b,c,d,e,f^	7.25±0.76
RG	108	10.46±1.01^a,e,f^	20.87±1.02^c,d,e,f^	62.91±1.11^a,c,d,e,f^	5.69±0.88
LC-0.3	110	10.12±0.77^a,e,f^	16.73±1.00^a,b c,d,e,f^	68.35±0.84^a,b,d,e,f^	4.71±0.55
LC-0.6	114	8.44±0.86^a,e,f^	15.19±0.77^a,b,c,f^	71.14±0.69^a,b,c,e,f^	5.16±0.90
CM-25%	110	5.37±0.91^a,b,c,d^	14.52±1.06^a,b,c,f^	75.22±1.01^a,b,c,d,f^	4.82±1.03
CM-50%	112	5.24±1.02^a,b,c,d^	10.23±0.65^a,b,c,d,e^	81.59±0.93^a,b,c,d,e^	4.14±0.88
Endometriosis					
Co	105	18.43±0.089^b,c,d,e,f^	35.53±0.015^e,f^	39.13±0.022^c,d,e,f^	5.40±0.011
RG	102	12.72±0.086^a,f^	38.77±0.008^e,f^	38.91±0.012^c,d,e,f^	9.22±0.016^c,d^
LC-0.3	107	12.52±0.066^a,f^	35.35±0.007^e,f^	50.45±0.025^a,b,e,f^	1.96±0.013^b,f^
LC-0.6	105	8.31±0.046^a^	35.21±0.004^e,f^	54.14±0.022^a,b,e,f^	3.34±0.023^b^
CM-25%	106	9.28±0.044^a^	16.25±0.011^a,b,c,d^	69.13±0.014^a,b,c,d^	4.63±0.028
CM-50%	104	7.14±0.018^a,b,c^	8.83±0.018^a,b,c,d,e^	76.86±0.009^a,b,c,d,e^	7.11±0.031^c^

Data are reported as means±SE. Co: Control; RG: Repaglinide at 1 µM; LC-0.3 and 0.6 mg/mL: LC: L-Carnitine at 0.3 and 0.6 mg/mL; CM-25%, -50%: Conditioned medium of bone marrow mesenchymal stem cells at 25 and 50%; GV: germinal vesicle; GVBD: germinal vesicle break down; MII: metaphase II; DEG: degenerated oocytes. ^a^P<0.05 compared with Co; ^b^P<0.05 compared with RG; ^c^P<0.05 compared with LC-0.3; ^d^P<0.05 compared with LC-0.6; ^e^P<0.05 compared with CM-25%; ^f^P<0.05 compared with CM-50% (chi-squared test).

Table 1 also provides IVM data from the EMS group. The 0.3 and 0.6 mg/mL LC, and 25 and 50% BMSC-CM enhanced the percentage of MII oocytes significantly. However, no considerable difference was observed between the control and RG groups. The RG, LC, and BMSC-CM significantly reduced the percentage of GV oocytes compared to control group.

### Assessment of nitro-oxidative stress

Table 2 shows the levels of TAC and NO in the normal and EMS groups. Our results revealed that 0.3 and 0.6 mg/mL LC and 25 and 50% BMSC-CM significantly decreased NO levels and significantly increased TAC levels compared to the control group. Yet, this significant alteration was not observed between the RG treatment and the control group. More notably, in both the normal and EMS groups, the highest TAC level was observed in the 0.6 mg/mL LC treatment.

**Table 2:** Assessment of antioxidant capacity in the normal and endometriosis groups.

Groups	NO (nmol/mg)	TAC (nmol/mg)
Normal		
Co	48.09±1.89^d,e,f^	0.97±0.093^c,d,e,f^
RG	50.00±1.31^c,d,e,f^	0.90±0.046^c,d,e,f^
LC-0.3	42.72±1.97^b^	1.22±0.079^a,b,d^
LC-0.6	39.58±1.09^a,b^	1.48±0.058^a,b,c^
CM-25%	39.92±1.49^a,b^	1.30±0.056^a,b^
CM-50%	38.76±1.51^a,b^	1.29±0.070^a,b^
Endometriosis		
Co	85.42±1.12^c,d,e,f^	0.52±0.022^c,d,e,f^
RG	88.34±1.36^c,d,e,f^	0.48±0.036^c,d,e,f^
LC-0.3	70.21±1.04^a,b^	0.74±0.046^a,b^
LC-0.6	62.38±1.2^a,b,e^	0.83±0.031^a,b,e^
CM-25%	71.45±1.26^a,b,d^	0.71±0.043^a,b,d^
CM-50%	66.34±1.40^a,b^	0.75±0.041^a,b^

Data are reported as means±SE. Co: Control; RG: Repaglinide at 1µM; LC-0.3, 0.6 mg/mL: L-Carnitine at 0.3 and 0.6 mg/mL; CM-25%, -50%: Conditioned medium of bone marrow mesenchymal stem cells at 25 and 50%; NO: nitric oxide levels; TAC: total antioxidant capacity. ^a^P<0.05 compared with Co; ^b^P<0.05 compared with RG; ^c^P<0.05 compared with LC-0.3; ^d^P<0.05 compared with LC-0.6; ^e^P<0.05 compared with CM-25%; ^f^P<0.05 compared with CM-50% (ANOVA).

### Evaluation of *in vitro* fertilization and embryo development

Table 3 shows the different stages of embryonic development in the normal groups. There was a substantial improvement in IVF, cleavage, and blastocyst rates in all treatment groups compared to the control group. in addition, the highest blastocyst formation rate was obtained after supplementation of IVM medium with a 50% BMSC-CM (73.19±0.82) (P<0.05). Table 3 also shows the results of *in vitro* embryo development in the EMS groups. A significant enhancement in IVF rate was observed in the 0.6 mg/mL LC and 25 and 50% BMSC-CM treatment groups, and there were significant differences in cleavage rate in all treatment groups compared to the control. Moreover, 0.3 and 0.6 mg/mL LC and 25 and 50% BMSC-CM exhibited a rise in blastocyst rate compared to the control group, and the highest blastocyst percentage was associated with 50% BMSC-CM (53.28±0.24).

**Table 3:** Percentage of different steps of mice embryo development in the normal experimental and endometriosis experimental groups.

Groups	MII (n)	IVF (%)	Cleavage (%)	Morula (%)	Blastocyst (%)	Degenerated (%)
Normal						
Co	120	72.66±1.13^b,c,d,e,f^	60.46±1.14^b,c,d,e,f^	7.72±1.01	47.82±0.66^b,c,d,e,f^	5.21±1.09
RG	124	78.74±1.10^a,d,e,f^	65.24±0.83^a,d,e,f^	4.31±0.95	54.48±0.52^a,d,e,f^	5.36±0.35
LC-0.3	122	79.16±0.96^a,e,f^	68.84±0.93^a,e,f^	4.16±1.17	59.11±1.04^a,d,e,f^	3.25±0.61
LC-0.6	126	85.33±1.07^a,b,f^	72.38±1.05^a,b,f^	5.28±0.91	63.16±0.74^a,b,c,f^	3.84±0.95
CM-25%	124	88.90±0.88^a,b,c^	77.56±0.75^a,b,c,f^	6.14±0.48	67.55±0.87^a,b,c^	3.78±0.74
CM-50%	122	93.77±0.92^a,b,c,d^	84.81±0.44^a,b,c,d,e^	7.20±0.62	73.19±0.82^a,b,c,d^	4.32±1.04
Endometriosis						
Co	134	70.00±0.25^d,e,f^	50.13±0.08^b,c,d,e,f^	10.23±0.03^b,c,d,e,f^	18.09±0.10^c,d,e,f^	13.14±0.17^d,e,f^
RG	130	71.32±0.04^d,e,f^	57.17±0.05^a,c,d,e,f^	16.14±0.17^a,d,e,f^	19.27±0.34^c,d,e,f^	11.06±0.19
LC-0.3	128	72.58±0.10^d,e,f^	63.54±0.20^a,b,e,f^	18.29±0.38^a,d^	26.33±0.11^a,b,d,e,f^	9.21±0.05
LC-0.6	131	81.20±0.05^a,b,c,f^	65.45±0.34^a,b,e,f^	23.19±0.18^a,b^	34.40±0.32^a,b,c,e,f^	8.05±0.14^a^
CM-25%	136	83.31±0.17^a,b,c,f^	73.25±0.11^a,b,c,d^	21.06±0.20^a,b^	45.05±0.19^a,b,c,d,f^	8.00±0.16^a^
CM-50%	132	91.59±0.0^a,b,c,d,e^	78.63±0.14^a,b,c,d^	24.59±0.41^a,b,c^	53.28±0.24^a,b,c,d,e^	8.04±0.11^a^

Data are reported as means±SE. Co: Control; RG: Repaglinide at 1 µM; LC-0.3, 0.6 mg/mL: L-Carnitine at 0.3 and 0.6 mg/mL; CM-25%, -50%: Conditioned medium of bone marrow mesenchymal stem cells at 25 and 50%; MII: metaphase II; IVF: *in vitro* fertilization rate. ^a^P<0.05 compared with Co; ^b^P<0.05 compared with RG; ^c^P<0.05 compared with LC-0.3; ^d^P<0.05 compared with LC-0.6; ^e^P<0.05 compared with CM-25%; ^f^P<0.05 compared with CM-50% (chi-squared test).

### Analysis of blastocyst quality

There was a dramatic increase in the mean total cell number and TE cells in the 1 µM RG, 0.3 and 0.6 mg/mL LC, and 25 and 50% BMSC-CM. More importantly, among all treated normal groups, only 50% BMSC-CM had a significant effect on ICM compared to the control group (P<0.05) (Table 4). Results of EMS groups are also reported in Table 4. Accordingly, except for the 1M RG group, all treatment groups exhibited a significant difference in the mean total cell population and TE cells compared to the control group. Nevertheless, there was no significant difference in blastocysts ICM among treated groups compared with the control group.

**Table 4:** Evaluation of blastocyst cell numbers in the normal experimental and the endometriosis experimental groups at 96 h post-*in vitro* fertilization.

Groups	Blastocysts (n)	Total cells (n)	TE (n)	ICM (n)
Normal				
Co	25	51.24±0.35^b,c,d,e,f^	37.82±0.61^c,d,e,f^	13.26±0.28
RG	25	56.33±0.44^a,e,f^	43.16±0.52^f^	13.04±0.61
LC-0.3	25	57.82±0.58^a,e,f^	44.77±0.94^a^	13.01±0.35
LC-0.6	25	59.36±0.48^a,f^	44.98±0.28^a^	14.30±0.73
CM-25%	25	62.95±0.88^a,b,c^	46.52±0.66^a^	16.41±0.80
CM-50%	25	65.87±0.56^a,b,c,d^	47.24±0.76^a,b^	18.57±0.25^a^
Endometriosis				
Co	25	47.12±0.91^c,d,e,f^	36.11±1.07^c,d,e,f^	10.90±0.39
RG	25	48.02±0.67^d,e,f^	36.24±0.78^c,d,e,f^	11.18±0.56
LC-0.3	25	52.75±1.04^a,f^	41.35±0.56^a,b^	11.33±0.42
LC-0.6	25	54.36±0.48^a,b^	41.94±0.28^a,b^	12.35±1.02
CM-25%	25	54.84±0.72^a,b^	43.78±0.44^a,b^	11.06±0.47
CM-50%	25	57.25±0.81^a,b,c^	44.89±0.93^a,b^	12.34±0.66

Data are reported as means±SE. Co: Control; RG: Repaglinide at 1 µM; LC-0.3, 0.6 mg/mL: L-Carnitine at 0.3 and 0.6 mg/mL; CM-25%, -50%: Conditioned medium of bone marrow mesenchymal stem cells at 25 and 50%; TE: trophectoderm; ICM: inner cell mass. ^a^P<0.05 compared with Co; ^b^P<0.05 compared with RG; ^c^P<0.05 compared with LC-0.3; ^d^P<0.05 compared with LC-0.6; ^e^P<0.05 compared with CM-25%; ^f^P<0.05 compared with CM-50% (ANOVA).

## Discussion

Many infertile women with EMS undergo IVF to increase their chances of achieving a pregnancy (25). However, in general, EMS is linked to low oocyte yield, implantation rates, and pregnancy rates following IVF (3). Among the factors associated with infertility in EMS women, oocyte quality is the most critical since it represents the intrinsic developmental potential and is responsible for proper fertilization/embryonic development during IVF (1,3). Surprisingly, limited studies have been conducted to examine the impacts of EMS on oocyte quality.

ROS has detrimental effects on oocytes and oxidative stress plays an important role in the pathogenesis of abnormal oocyte development (26). In accordance with this, our current findings indicated that EMS induction led to a considerable drop in TAC levels of the IVM medium. Since TAC is the result of the interactions among its numerous components, it reflects the potential to protect against free radical damage more effectively than individual plasma antioxidant measurements (11). Similarly, EMS also causes severe impairment in the generation and metabolism of NO (27). NO is a ubiquitous free radical in the oocyte microenvironment involved in the physiology and biology of the ovary and every stage of oocyte development, including meiotic maturation, fertilization, embryonic cleavage, and implantation (27). As a result of diminished bioavailability of NO under certain pathologic conditions, oocyte viability and developmental capacity may be compromised (27). In this regard, NO oxidation by O_2_·- produces peroxynitrite (ONOO-), a highly reactive molecule that depletes lipid-soluble antioxidants, contributing to oxidative stress and lipid peroxidation in the oocyte microenvironment, which mediates an adverse impact on oocyte quality (27). Our data support the results of previous animal experiments and human trials showing that the level of NO in the IVM medium was significantly raised in EMS model groups, which could reflect nitrosative stress. Therefore, to preserve follicles from oxidative damage, the follicular fluid is naturally provided with an effective antioxidant system comprised of enzymatic antioxidants and vitamins (28). It is important to note that *in vitro* environmental conditions such as increased exposure to oxygen, light, and culture medium composition trigger metabolic alterations in oocytes and embryos, resulting in an imbalance between the ROS formation and antioxidant capacity (29). Thus, adding anti-oxidative components to the IVM medium of EMS subjects is likely to provide more appropriate conditions and boost maturation, fertilization, and further embryo development (29).

We recently discovered that supplementing IVM medium with RG promotes oocyte maturation and embryo cleavage rate by elevating the intracellular calcium concentration (9). In line with this, the present findings revealed that RG significantly improved nuclear oocyte maturation in normal mice. More importantly, the rates of fertilization, cleavage, and blastulation were positively changed in the RG-supplemented normal mice. Since alterations of the oocyte cytoskeleton have been documented to be one of the reasons for poor oocyte quality in EMS subjects, we assumed that RG may reverse this impact by raising intracellular calcium concentration (1,3). In addition, dysregulation of intracellular Ca^2+^ concentration with resulting poor oocyte quality has been recently attributed to oxidative stress in oocytes and their microenvironment (30). Accordingly, exposure to ROS might be a primary cause of abnormal patterns of Ca^2+^ release at fertilization (4). Thus, RG could combat this phenomenon by increasing intracellular calcium concentration. However, when determining the percentages of GV, GVBD, and MII oocytes in the EMS-induced groups, our results demonstrated that the addition of RG did not affect the rates of nuclear oocyte maturation compared to the control group. Similarly, adding RG to the IVM medium did not improve fertilization rates and embryo development. Even though some recent studies claim that RG possesses anti-oxidative properties and significantly affects lipid peroxidation levels in an *in vivo* study, our results indicated that RG did not improve antioxidant status in IVM medium (31). In other words, the levels of TAC and NO in the IVM medium were not significantly affected by RG in both normal and EMS groups.

Previous research reported an EMS-dependent decline in oocyte quality attributed to the improper energy metabolism of fatty acids and/or the mitochondrial dysfunction detected in the oocytes and cumulus oophorus cells of EMS women (32). On the contrary, LC can facilitate fatty acid and energy application by transporting long-chain fatty acids through the inner mitochondrial membrane for β-oxidation, subsequently increasing the concentration of adenosine triphosphate (ATP) (32). Interestingly, the β-oxidation process is essential in the nuclear and cytoplasmic maturation of oocytes, leading to oocyte developmental competence (33). The dual role of LC as an antioxidant and as an important element of lipid metabolism makes it an option as a novel non-invasive agent for optimizing oocyte competence efficiency and subsequent embryonic development (34). In this regard, the obtained results indicated that the treatment of normal and EMS-induced immature oocytes with LC during IVM increased the proportion of oocytes that reached the MII stage and reduced oocyte degeneration rate. These results are consistent with prior canine and porcine studies demonstrating that adding LC to the IVM medium improved nuclear maturation and subsequent embryo development following IVF (35,36). In addition, here, LC improved cleavage and blastocyst rates as well as total blastocyst cell numbers when added to the maturation medium of EMS-induced and EMS-free mice. In line with this, LC supplementation (1.5-3 mM) to embryo culture enhanced lipid metabolism in bovine embryos, most likely by β-oxidation and ATP production, leading to improved blastocyst development and blastocyst cell numbers (37). In this context, Jiang et al. (38) reported that supplementation of the IVC medium with LC enhanced the development of zygotes from bovine aged oocytes to the blastocyst stage, as well as the quality of the blastocysts. Our results also showed that LC elevated TAC levels in maturation medium, which is in accordance with a recent study that indicated that supplementing IVM medium with 0.5 mg/mL LC significantly increased intracellular GSH levels of porcine matured oocytes and improved development competence of parthenogenetic embryos (36). This effect was attributed to the effect of LC on ROS and thus preserving GSH reserves in porcine mature oocytes. Moreover, LC supplementation has been reported to boost the activities of antioxidant enzymes, including superoxide dismutase, catalase, and glutathione peroxidase, which constitute a natural defense system against oxidant activity (38). Consistently, our findings revealed that LC was the most effective supplement for enhancing TAC levels in the IVM medium among all treated groups.

As shown previously, MSCs secrete various cytokines and growth factors into MSC-CM that can improve *in vitro* meiotic maturation and subsequent embryonic developmental potential (18). In the current study, the co-culture of normal and EMS-induced immature oocytes with BMSC-CM improved the quality of the medium and IVM and IVF rates and increased the rates of blastocyst production compared to the control group. Our results agree with the study of Ling et al. (39) in which the maturation rate of mouse oocytes was higher in MSC-CM compared to that in the control group. Indeed, recent studies have established that MSC-CM contains a variety of cytokines, growth factors, and anti-apoptotic and antioxidant components that may help in the maintenance of IVM and fertilization rates that are comparable to those observed in the control group (17,18). In our findings, treatments containing BMSC-CM also increased TAC levels while diminishing NO levels in the IVM medium. The bioactive factors of MSC-CM have the potential to modulate oxidative stress by decreasing ROS and boosting the expression of antioxidant enzymes (17). Furthermore, a Ca^2+^ increase is an early detectable indicator of oocytes activation, stimulating the resumption of meiosis and the formation of pronuclear (40). It is noteworthy that MSC-CM can operate as an effective parthenogenetic agent, mimicking the critical events of oocyte activation, including Ca^2+^ elevation, meiosis resumption, pronuclear formation, and parthenogenetic development (40). To sum up, we demonstrated that 25 and 50% BMSC-CM supplementation during IVM improved maturation, fertilization, and the subsequent development of EMS-induced oocytes. We also showed that 50% BMSC-CM was the most beneficial concentration to be used. In fact, it resulted in higher maturation and embryo developmental rates than the control and other treated groups. However, one of the study limitations was that we did not measure the actual levels of growth factors, pro-inflammatory cytokines, anti-apoptotic agents, and antioxidants in the IVM medium. We, therefore, do not know which of the constituents of the BMSC-CM in particular had promoter effects on the oocyte maturation, fertilization, and developmental competence of IVF embryos.

In conclusion, although the relevance of the results here obtained is limited by the use of an animal model, we demonstrated for the first time that supplementing endometriosis-induced oocytes with LC and BMSC-CM during IVM improved their maturation and fertilization rates and subsequent preimplantation embryo development following IVF and embryo culture. Among the different supplementations and concentrations examined, 50% BMSC-CM seemed to be the most beneficial one, as it resulted in higher rates of morula development on day 5. These novel approaches may have clinical applications in the ARTs setting and may improve fertility outcomes in endometriosis-related infertile couples. Nonetheless, more studies are required to determine the precise molecular and subcellular mechanisms underlying the role of RG, LC, and BMSC-CM in oocyte maturation and embryo development of endometriosis-derived oocytes.

## Supplementary material

Click to view [pdf].

## References

[1] Xu B, Guo N, Zhang XM, Shi W, Tong XH, Iqbal F (2015). Oocyte quality is decreased in women with minimal or mild
endometriosis. Sci Rep.

[2] Shebl O, Sifferlinger I, Habelsberger A, Oppelt P, Mayer RB, Petek E (2017). Oocyte competence in *in vitro* fertilization and
intracytoplasmic sperm injection patients suffering from endometriosis and
its possible association with subsequent treatment outcome: a matched
case-control study. Acta Obstet Gynecol Scand.

[3] Sanchez AM, Vanni VS, Bartiromo L, Papaleo E, Zilberberg E, Candiani M (2017). Is the oocyte quality affected by endometriosis? A review of the
literature. J Ovarian Res.

[4] Agarwal A, Aponte-Mellado A, Premkumar BJ, Shaman A, Gupta S (2012). The effects of oxidative stress on female reproduction: a
review. Reprod Biol Endocrinol.

[5] Hamdan M, Dunselman G, Li T, Cheong Y (2015). The impact of endometrioma on IVF/ICSI outcomes: a systematic
review and meta-analysis. Hum Reprod Update.

[6] Moradi M, Karimi I, Ahmadi S, Mohammed LJ (2020). The necessity of antioxidant inclusion in caprine and ovine semen
extenders: a systematic review complemented with computational
insight. Reprod Domest Anim.

[7] Scutiero G, Iannone P, Bernardi G, Bonaccorsi G, Spadaro S, Volta CA (2017). Oxidative stress and endometriosis: a systematic review of the
literature. Oxid Med Cell Longev.

[8] Li C, Choi DH, Choi JS (2012). Effects of efonidipine on the pharmacokinetics and
pharmacodynamics of repaglinide: possible role of CYP3A4 and P-glycoprotein
inhibition by efonidipine. J Pharmacokinet Pharmacodyn.

[9] Kalehoei E, Azadbakht M (2017). The beneficial effect of repaglinide on *in vitro*
maturation and development ability of immature mouse oocytes. In vitro Cell Dev Biol Anim.

[10] Tankova T, Koev D, Dakovska L, Kirilov G (2003). The effect of repaglinide on insulin secretion and oxidative
stress in type 2 diabetic patients. Diabetes Res Clin Pract.

[11] Gumieniczek A, Hopkała H, Roliński J, Bojarska-Junak A (2005). Antioxidative and anti-inflammatory effects of repaglinide in
plasma of diabetic animals. Pharmacol Res.

[12] Longo N, Frigeni M, Pasquali M (2016). Carnitine transport and fatty acid oxidation. Biochim Biophys Acta Mol Cell Res.

[13] Gnoni A, Longo S, Gnoni GV, Giudetti AM (2020). Carnitine in human muscle bioenergetics: can carnitine
supplementation improve physical exercise?. Molecules.

[14] Terruzzi I, Montesano A, Senesi P, Villa I, Ferraretto A, Bottani M (2019). L-Carnitine reduces oxidative stress and promotes cells
differentiation and bone matrix proteins expression in human osteoblast-like
cells. Biomed Res Int.

[15] Moawad AR, Tan SL, Xu B, Chen HY, Taketo T (2013). L-carnitine supplementation during vitrification of mouse oocytes
at the germinal vesicle stage improves preimplantation development following
maturation and fertilization *in vitro*. Biol Reprod.

[16] Khan R, Jiang X, Hameed U, Shi Q (2021). Role of lipid metabolism and signaling in mammalian oocyte
maturation, quality, and acquisition of competence. Front Cell Dev Biol.

[17] Bezerra MÉS, Monte APO, Barberino RS, Lins TLBG, Oliveira JL, Santos JMS (2019). Conditioned medium of ovine Wharton's jelly-derived mesenchymal
stem cells improves growth and reduces ROS generation of isolated secondary
follicles after short-term *in vitro* culture. Theriogenology.

[18] Akbari H, Vaghefi SHE, Shahedi A, Habibzadeh V, Mirshekari TR, Ganjizadegan A (2017). Mesenchymal stem cell-conditioned medium modulates apoptotic and
stress-related gene expression, ameliorates maturation and allows for the
development of immature human oocytes after artificial
activation. Genes (Basel).

[19] Somigliana E, Viganò P, Rossi G, Carinelli S, Vignali M, Panina-Bordignon P (1999). Endometrial ability to implant in ectopic sites can be prevented
by interleukin-12 in a murine model of endometriosis. Hum Reprod.

[20] Zhaleh H, Azadbakht M, Pour AB (2016). Protective effects of mouse bone marrow mesenchymal stem cell
soup on staurosporine induced cell death in PC12 and U87 cell
lines. Int J Med Res Health Sci.

[21] Almohammed ZNH, Moghani-Ghoroghi F, Ragerdi-Kashani I, Fathi R, Tahaei LS, Naji M (2020). The effect of melatonin on mitochondrial function and autophagy
in *in vitro* matured oocytes of aged mice. Cell J.

[22] Moradi M, Goodarzi N, Faramarzi A, Cheraghi H, Hashemian AH, Jalili C (2021). Melatonin protects rats testes against bleomycin, etoposide, and
cisplatin-induced toxicity via mitigating nitro-oxidative stress and
apoptosis. Biomed Pharmacother.

[23] Giritharan G, Talbi S, Donjacour A, Di Sebastiano F, Dobson AT, Rinaudo PF (2007). Effect of *in vitro* fertilization on gene
expression and development of mouse preimplantation embryos. Reproduction.

[24] Maylem ERS, Leoveras MED, Atabay EC, Atabay EP (2017). Assessing the quality of bovine embryos produced *in
vitro* through the inner cell mass and trophectoderm
ratio. Philipp Jof Sci.

[25] Bulletti C, Coccia ME, Battistoni S, Borini A (2010). Endometriosis and infertility. J Assist Reprod Genet.

[26] Wang S, He G, Chen M, Zuo T, Xu W, Liu X (2017). The role of antioxidant enzymes in the ovaries. Oxid Med Cell Longev.

[27] Goud PT, Goud AP, Joshi N, Puscheck E, Diamond MP, Abu-Soud HM (2014). Dynamics of nitric oxide, altered follicular microenvironment,
and oocyte quality in women with endometriosis. Fertil Steril.

[28] Luddi A, Capaldo A, Focarelli R, Gori M, Morgante G, Piomboni P (2016). Antioxidants reduce oxidative stress in follicular fluid of aged
women undergoing IVF. Reprod Biol Endocrinol.

[29] Agarwal A, Durairajanayagam D, du Plessis SS (2014). Utility of antioxidants during assisted reproductive techniques:
an evidence based review. Reprod Biol Endocrinol.

[30] Mihalas BP, Redgrove KA, McLaughlin EA, Nixon B (2017). Molecular mechanisms responsible for increased vulnerability of
the ageing oocyte to oxidative damage. Oxid Med Cell Longev.

[31] Obi BC, Okoye TC, Okpashi VE, Igwe CN, Alumanah EO (2016). Comparative study of the antioxidant effects of metformin,
glibenclamide, and repaglinide in alloxan-induced diabetic
rats. J Diabetes Res.

[32] Agarwal A, Sengupta P, Durairajanayagam D (2018). Role of L-carnitine in female infertility. Reprod Biol Endocrinol.

[33] Dunning KR, Cashman K, Russell DL, Thompson JG, Norman RJ, Robker RL (2010). Beta-oxidation is essential for mouse oocyte developmental
competence and early embryo development. Biol Reprod.

[34] Fathi M, El-Shahat K (2017). L-carnitine enhances oocyte maturation and improves *in
vitro* development of embryos in dromedary camels
(*Camelus dromedaries*). Theriogenology.

[35] Moawad AR, Salama A, Badr MR, Fathi M (2021). Beneficial effects of L-carnitine supplementation during IVM of
canine oocytes on their nuclear maturation and development *in
vitro*. Animals (Basel).

[36] Somfai T, Kaneda M, Akagi S, Watanabe S, Haraguchi S, Mizutani E (2011). Enhancement of lipid metabolism with L-carnitine during
*in vitro* maturation improves nuclear maturation and
cleavage ability of follicular porcine oocytes. Reprod Fertil Dev.

[37] Takahashi T, Inaba Y, Somfai T, Kaneda M, Geshi M, Nagai T (2013). Supplementation of culture medium with L-carnitine improves
development and cryotolerance of bovine embryos produced *in
vitro*. Reprod Fertil Dev.

[38] Jiang W, Li Y, Zhao Y, Gao Q, Jin Q, Yan C (2020). L-carnitine supplementation during *in vitro*
culture regulates oxidative stress in embryos from bovine aged
oocytes. Theriogenology.

[39] Ling B, Feng D, Zhou Y, Gao T, Wei H, Tian Z (2008). Effect of conditioned medium of mesenchymal stem cells on the
*in vitro* maturation and subsequent development of mouse
oocyte. Braz J Med Biol Res.

[40] Feng D, Zhou Y, Ling B, Gao T, Shi Y, Wei H (2009). Effects of the conditioned medium of mesenchymal stem cells on
mouse oocyte activation and development. Braz J Med Biol Res.

